# Experimental Evolution of a Novel Sexually Antagonistic Allele

**DOI:** 10.1371/journal.pgen.1002917

**Published:** 2012-08-30

**Authors:** Rebecca Dean, Jennifer C. Perry, Tommaso Pizzari, Judith E. Mank, Stuart Wigby

**Affiliations:** 1 Department of Zoology, Edward Grey Institute, Oxford, United Kingdom; 2 Department of Evolutionary Biology, Evolutionary Biology Centre, Uppsala University, Uppsala, Sweden; 3 Department of Genetics, Evolution, and Environment, University College London, London, United Kingdom; University of California Davis, United States of America

## Abstract

Evolutionary conflict permeates biological systems. In sexually reproducing organisms, sex-specific optima mean that the same allele can have sexually antagonistic expression, i.e. beneficial in one sex and detrimental in the other, a phenomenon known as intralocus sexual conflict. Intralocus sexual conflict is emerging as a potentially fundamental factor for the genetic architecture of fitness, with important consequences for evolutionary processes. However, no study to date has directly experimentally tested the evolutionary fate of a sexually antagonistic allele. Using genetic constructs to manipulate female fecundity and male mating success, we engineered a novel sexually antagonistic allele (SAA) in *Drosophila melanogaster.* The SAA is nearly twice as costly to females as it is beneficial to males, but the harmful effects to females are recessive and X-linked, and thus are rarely expressed when SAA occurs at low frequency. We experimentally show how the evolutionary dynamics of the novel SAA are qualitatively consistent with the predictions of population genetic models: SAA frequency decreases when common, but increases when rare, converging toward an equilibrium frequency of ∼8%. Furthermore, we show that persistence of the SAA requires the mating advantage it provides to males: the SAA frequency declines towards extinction when the male advantage is experimentally abolished. Our results empirically demonstrate the dynamics underlying the evolutionary fate of a sexually antagonistic allele, validating a central assumption of intralocus sexual conflict theory: that variation in fitness-related traits within populations can be maintained via sex-linked sexually antagonistic loci.

## Author Summary

Males and females are markedly different in many features, meaning that a trait that is beneficial for one sex may be detrimental for the other. Recent studies show that this type of sexual antagonism is abundant in natural populations; however, no study has tested the evolutionary fate of a sexually antagonistic allele. Using genetic manipulations to alter female fecundity and male mating success, we generated a novel sexually antagonistic allele in *Drosophila melanogaster*, allowing us to study whether such an allele can persist in populations. We show that the sexually antagonistic allele causes more harm to females than it provides benefits to males but—as predicted by theory—it is able to persist in the population. This is because the harmful effects to females are both recessive (it is only harmful when two copies of the allele are present) and linked to the X-chromosome, so females are rarely harmed when the allele is at low frequency. These results show how a sexually antagonistic allele can be maintained in populations and contribute to maintain variation in male and female reproductive success.

## Introduction

Understanding the mechanisms that promote variation in fitness-related traits within populations presents an enduring challenge in evolutionary biology [1], [2]: intralocus sexual conflict is predicted to be one such mechanism [3]–[6]. Intralocus conflict occurs when the same allele at a single locus provides net fitness benefits when expressed in one sex but net fitness costs when expressed in the other [7]. Although this conflict can potentially be resolved by the evolution of sexual dimorphism [8], a growing body of studies provide evidence that substantial sexually antagonistic variation occurs in both natural [9], [10] and laboratory-adapted populations [11]–[18]. To date, the main approaches used to identify the presence of intralocus sexual conflict have been the detection of negative genetic correlations for fitness between males and females [9]–[17] and experimental evolution using sex-limited selection [14], [19]. These studies have highlighted the extent to which sexually antagonistic selection affects fitness-related traits, and have identified candidate sexually antagonistic genes. However, no previous empirical studies have characterized the evolutionary dynamics of a specific sexually antagonistic allele.

We aimed to validate predictions made by intra-locus sexually antagonistic theory by experimentally engineering a novel sexually antagonistic X-linked allele. We empirically explored a fundamental principle of intralocus sexual conflict theory: that a recessive allele that benefits the heterogametic sex but harms the homogametic sex can invade a population, even when the cost exceeds the benefit, if the locus is located on the homogametic sex-chromosome [6]. This prediction arises because at low population frequency the costly effects of the allele for the homogametic sex are limited to homozygotes, which are rare, whereas the benefits are always expressed in the hemizygous sex. Consequently, such an allele could theoretically invade and reach an equilibrium frequency [6]. This makes the X-chromosome a potential hot spot for such sexually antagonistic genetic variation [20] and thus an ideal target for intralocus sexual conflict research.

We first used genetic manipulations to generate a putative sexually antagonistic allele on the X-chromosome of *Drosophila melanogaster*. We then tested: a) the magnitude of the cost to females (in terms of offspring production) and benefits to males (in terms of mating success), b) whether the allele could invade and persist in a population and how the invasion dynamics compared to predictions derived from theoretical models, and c) whether the evolutionary persistence of the allele was dependent upon the benefit provided to males.

## Results/Discussion

### Generation of a Novel Sexually Antagonistic Locus

**Figure 1:**
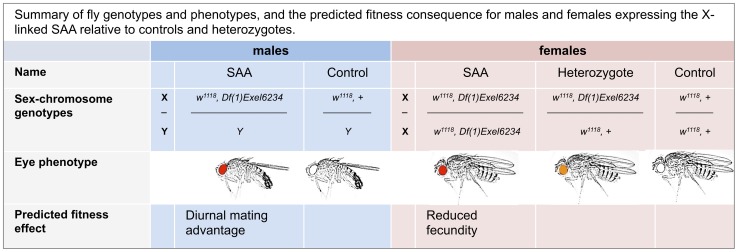
Summary of fly genotypes and phenotypes, and the predicted fitness consequence for males and females expressing the X-linked SAA (sexually antagonistic allele) relative to controls and heterozygotes.

To create a novel sexually antagonistic allele on the *D. melanogaster* X chromosome, we used two genetic constructs: 1) *Df(1)Exel6234*, a genetic deficiency which covers the *sex-peptide receptor* gene and 4 other genes of unknown function [21] and 2) *w^1118^*, a loss of function allele for the *white* gene which determines eye color [22]. Both *Df(1)Exel6234* and *w^1118^* are located on the X-chromosome. Homozygous *Df(1)Exel6234* females fail to react to the male seminal protein, sex peptide [23], and show reduced levels of sex-peptide-induced post-mating responses. For example, *Df(1)Exel6234* females lay significantly fewer eggs after mating than wild-type females [21]. Flies lacking *white* have white eyes, and white-eyed males suffer from impaired vision and reduced mating success compared to wild-type males (which have red eyes) in photophase (i.e., the light) [24], but not in the scotophase (i.e., the dark) [25]. In contrast, females lacking *white* suffer no obvious reduction in adult fitness (i.e., lifespan, fecundity or fertility) under standard laboratory conditions [26]. The *Df(1)Exel6234* deficiency carries a *white*+ transgene [27], which provides a partial rescue of *white* mutations (i.e., red eyes and improved vision). Tight linkage between the *Df(1)Exel6234* deficiency and the *white*+ transgene ensures that recombination between them is negligible. Thus, in a *w^1118^* background, male hemizygote and female homozygote carriers of *Df(1)Exel6234* possess red eyes, whilst heterozygote females possess orange eyes (Figure 1).

We confirmed that red-eyed *Df(1)Exel6234* bearing males have increased competitive mating success relative to *w^1118^* white-eyed males in photophase, presumably due to improved vision. In direct, one-on-one, male-male competition, *Df(1)Exel6234* bearing males were significantly more likely to achieve the first mating with a single virgin female in photophase (26/28 trials, binomial test, p<0.0001) but not in scotophase (winning 14/28 trials, binomial test, p = 0.57). We also tested whether the SAA has an effect on male post-copulatory competitive ability. Female *D. melanogaster* mate multiply [28] resulting in sperm competition [29], [30], and variation in sperm competitive ability can potentially have major impacts on male fitness [31], [32]. However, we found no significant differences in the sperm defense (P_1_) or sperm offense (P_2_) abilities of SAA and control males (P_1_ assay, Z = 1.145, P = 0.252; P_2_ assay, Z = 0.247, P = 0.805; Figure S1A and S1B).

**Figure 2:**
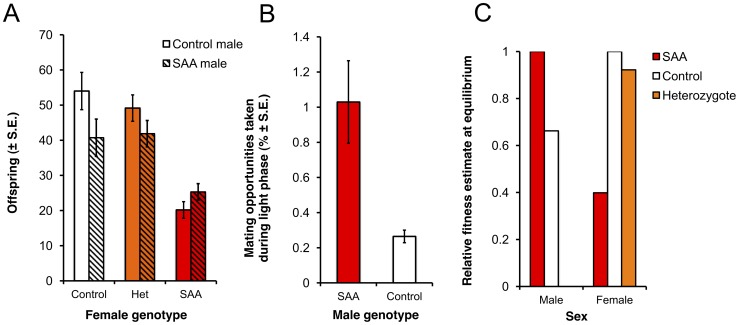
Reproductive success of male and female genotypes. (a) Homozygote sexually antagonistic allele (SAA) females suffer reproductive success costs compared to control and heterozygote females (F_2,168_ = 55.4, p<0.0001). Furthermore, reproducing with control males rather than SAA-males exacerbates the relative cost to SAA-female reproductive success (male*female: F_2,168_ = 5.27, p = 0.07). (b) SAA-males have a photophase mating advantage over control males in P4-P4 (χ^2^_1_ = 35.58, p<0.0001) (c) Estimates of relative fitness at the SAA equilibrium frequency (12.6%) for males and females of different genotypes. Relative fitness is calculated from the population genetic model for a 12∶12 light∶dark cycle. Note that the relative fitness of males is adjusted for scotophase, during which time the mating success of SAA and control males is equal. Therefore, the overall advantage to SAA males is lower than in photophase only (as shown in b) and the predicted fitness cost of SAA to homozygote females exceeds the predicted fitness benefit of SAA to males.

As expected, homozygous *Df(1)Exel6234* females suffer significant reproductive costs compared to heterozygote and control females (Figure 2a, Table S1). Thus, in a *w^1118^* background population, *Df(1)Exel6234* fits the conditions required for an X-linked sexually antagonistic allele: it benefits one sex but harms the other. Moreover, the costs of *Df(1)Exel6234* to females are recessive: we detected no significant fecundity cost to heterozygote females (Figure 2a, Tables S1, S2). We hereafter refer to individuals carrying the deficiency *Df(1)Exel6234* as the SAA (sexually antagonistic allele) flies and non-carriers as controls (Figure 1). All experimental flies carry *w^1118^*. We predicted that selection favouring the SAA males should drive the SAA allele to higher frequency in populations when it is rare, whilst selection against the SAA homozygote females should drive the SAA frequency down when it is common.

### Experimental Evolution and Modeling of a Novel Sexually Antagonistic Locus

To test the evolutionary fate of the male-beneficial, female-detrimental SAA, we simultaneously set up four replicate experimental populations (P1–P4) containing a mixture of SAA and control individuals. We initiated the populations with a SAA frequency of 3% and tracked the frequency of SAA for 16 generations in P1–P4, and a further 7 generations in two of these populations that we randomly selected (P1 and P2). Populations were maintained on a 12∶12 light dark cycle, and thus for 50% of the time (during the photophase), SAA males were predicted to possess a mating advantage (*D. melanogaster* mating activity occurs slightly more frequently in the dark [33], [34] when the mating advantage of SAA males is absent). We observed matings in P1–P4 during photophase over multiple generations, allowing us to estimate the relative mating fitness of SAA- *versus* control males in the population cage environment. We found that, as expected, SAA-males possessed a significant mating advantage in P1–P4 during photophase (Figure 2b).

Using these male mating frequency estimates (and assuming equal mating success between SAA and control males during scotophase), together with the expected mating rates during light *vs* dark phases [33], [34] and the genotype-specific frequencies of offspring produced from each type of cross (Table S1), we generated quantitative predictions for the spread and equilibrium of the SAA based on Rice's population genetic model [6]. Parameterizing the model with these data leads to the prediction that, over evolutionary time, the SAA should reach an equilibrium frequency at which the fitness cost to homozygote SAA females will exceed the fitness benefits to SAA-males (Figure 2c).

**Figure 3:**
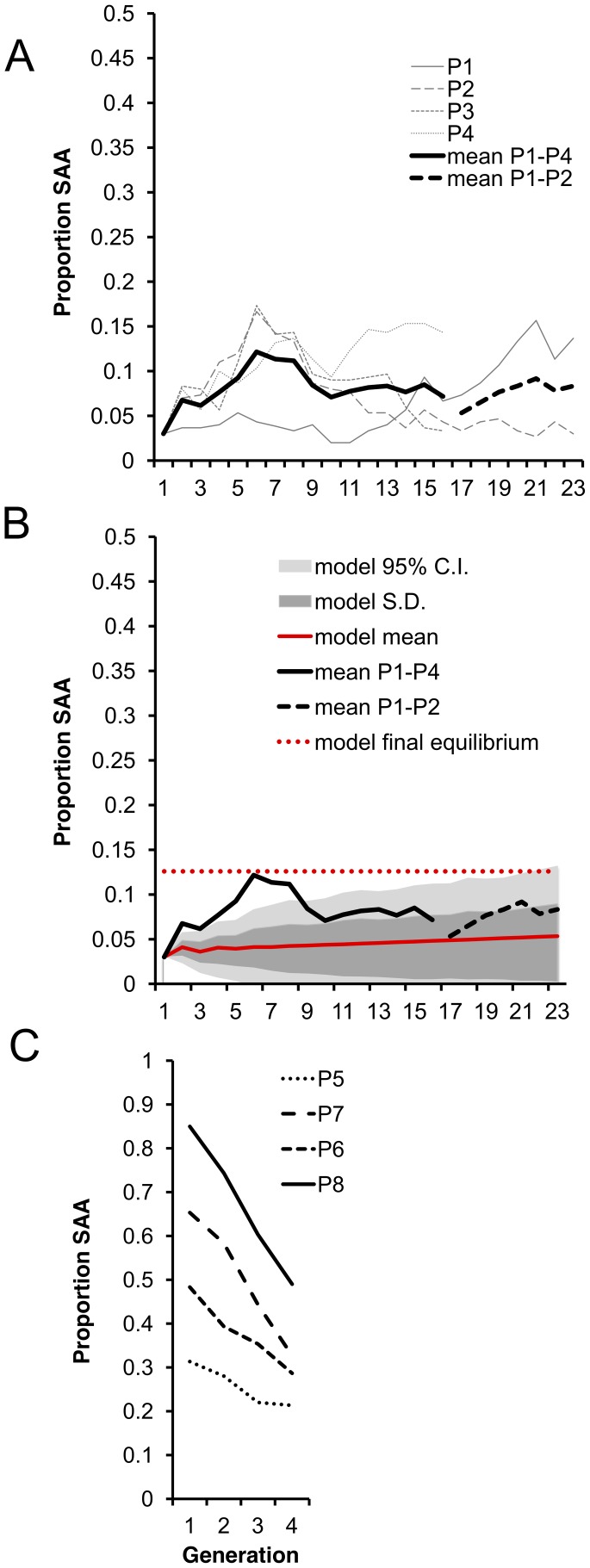
Experimental evolution of the SAA. (a) Mean SAA frequency changed significantly over the 16 experimental generations in a log-linear manner (ln linear term, χ^2^_1_ = 94.1, p<0.0001, linear term, χ^2^_1_ = 0.30, p = 0.58). SAA frequency increased significantly from the 1^st^ to 2^nd^ generation (χ^2^_1_ = 6.07, p = 0.014), indicating that the SAA bearing males had high fitness relative to controls (SAA was present only in males in the 1^st^ generation) and confirming that SAA frequency increases when rare. Segmented regressions showed that mean SAA frequency continued to increase until generation 6 (change from generation 1–6, χ^2^_1_ = 6.71, p = 0.0096) reaching ∼12%. SAA frequency then underwent a significant decline to ∼8% at generation 10 (change from generation 6–10, χ^2^_1_ = 5.14, p = 0.023) and thereafter did not change significantly in frequency (change from generation 10–16, χ^2^_1_ = 0.013, p = 0.91). Populations P1 and P2 only were maintained for generations 17–23. (b) The model (red solid line) predicted a steady increase in SAA frequency until an equilibrium frequency of 12.6% after 700 generations (red dashed line). The range of values expected from the model is shown by the 95% confidence limits (light grey area). (c) SAA frequency declined over 4 generations for each of the P5–8 populations (χ^2^_1_ = 10.89, p = 0.001). There was a significant interaction between the initial SAA frequency and generation, showing that the higher the initial SAA frequency, the further it declined (χ^2^_1_ = 11.049, p = 0.0009).

As predicted, average SAA frequency in P1–P4 significantly increased from the 3% starting frequency and appeared to reach a plateau at an equilibrium frequency. Initially, the frequency increased more rapidly than predicted by the model but thereafter stabilized around 8% (Figure 3a), which broadly agrees with the model predictions over the first 23 generations (Figure 3b). The model predicts an ultimate equilibrium of 12.6% (0.05–0.20 95% CI) after 700 generations, suggesting that over the 23 generations we measured, the SAA may not have reached its final equilibrium frequency.

To test the prediction that, due to the harmful effects on female fecundity, the SAA frequency should decline if the SAA is common, we set up a further 4 populations (P5–P8) with a range of higher initial SAA frequencies (31% to 85%) and measured SAA frequency over 3 subsequent generations. As expected, SAA frequency significantly declined in P5–P8. Moreover, the steepness of the decline was significantly greater in populations with higher initial frequencies (Figure 3c), confirming that SAA cannot be maintained at high frequencies, and suggesting that – regardless of the original frequency – SAA tends to converge towards a single stable equilibrium.

### SAA Persistence Is Dependent upon the Male Mating Advantage

**Figure 4:**
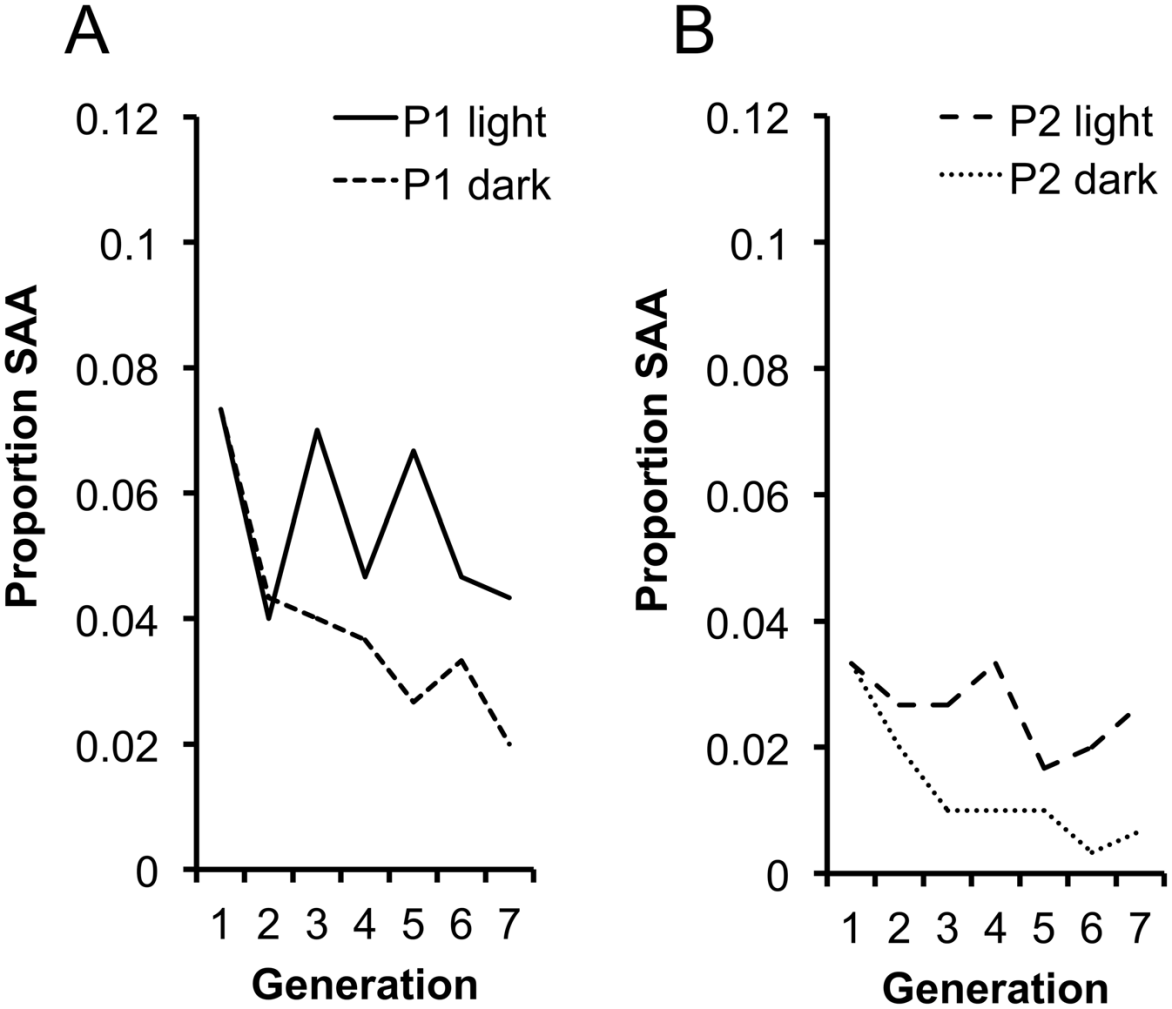
Changes in SAA frequency in the light and dark populations. SAA frequency was affected by the manipulation of light/dark regimes (χ^2^_1_ = 18.82, p<0.0001) across (a) P1 light and dark populations and (b) P2 light and dark populations. There was a significant interaction between light treatment and generation (χ^2^_1_ = 4.54, p = 0.033) showing that SAA frequency significantly diverged between the continuous light and continuous dark populations. SAA frequency did not significantly change in light populations (χ^2^_1_ = 2.97, p = 0.085) but significantly declined in dark populations (χ^2^_1_ = 4.81, p = 0.028).

A central assumption of our hypothesis is that the SAA invades, and is maintained in the population, as a result of the mating advantage it provides males during photophase. Without this advantage, we expect a decline in the SAA and eventual extinction due to the costs imposed upon SAA females. To test this prediction we set up replicate populations of P1 and P2 at generation 16 (in which the SAA frequencies were 0.073 and 0.033, respectively) and maintained adults in these populations in permanent dark (P1 dark, P2 dark) conditions, under which SAA males should posses no mating advantage. To control for the disruption to circadian rhythm we set up replicate control populations maintained in permanent light (P1 light, P2 light). We measured SAA frequency over 6 subsequent generations in the dark and light populations. As expected, within each replicate SAA frequency significantly decreased in the dark population relative to the light population (Figure 4a and 4b) indicating that the SAA male mating advantage in photophase is essential for the maintenance of SAA. Surprisingly, SAA did not increase in light populations, suggesting that additional hours of light did not provide significant additional fitness benefits to SAA males over the standard 12∶12 light∶dark conditions. Male *Drosophila* require scotophases to initiate courtship efficiently [35], therefore courtship and mating in SAA males might have been negatively affected by permanent light. Additionally, there may be constraints on male courtship rates, mating rates or ejaculate production that set an upper limit to SAA male reproductive capacity. Nevertheless, the results provide support for the hypothesis that SAA persists in populations as a result of the mating advantage it provides males during photophase.

### Experimental Support for Intralocus Sexual Conflict Theory

Our experimental data indicate that 1) SAA frequency declines when it is common, because there is a large negative impact on the fecundity of homozygous females 2) SAA persists in populations because of the mating benefit it provides males in photophase, and SAA frequency declines towards extinction if the mating advantage of SAA males is abolished and 3) SAA has a single equilibrium frequency that is of broadly similar magnitude to that predicted by models based on intra-locus sexual conflict theory. Quantitative discrepancies between the model and our empirical data – for example, the surprisingly rapid increase in SAA frequency in the P1–4 lines – may derive from a range of factors. For example, any potential subtle effects of the *Df(1)Exel6234* deficiency that have not been characterized – on development time, ejaculate depletion rates or other traits that might impact male or female fitness – might contribute to differences between model predictions and our observed SAA frequencies. Nevertheless, our results provide robust qualitative support for sexually antagonistic evolution.

### Conclusion

Previous empirical evidence for intralocus sexual conflict derives from studies that demonstrate negative intersexual correlations for fitness, sexually antagonistic selection on phenotypes, or changes in sexually dimorphic traits under sex-limited evolution (reviewed in reference [4]). Here we provide direct experimental support for the idea that that sexually antagonistic alleles can invade and persist in populations. Thus, our work provides a novel demonstration that – as predicted by theory – evolution can maintain fitness variation within populations via sex chromosome-linked sexually antagonistic alleles.

## Materials and Methods

### General Fly Methods

The control, white-eyed *white* ^Dahomey^, stock [36] was generated by repeatedly backcrossing *w* ^1118^ into the Dahomey wild-type background (>7 generations). *Df(1)Exel6234* [21] was backcrossed for 5 generations into *white* ^Dahomey^ to generate SAA flies. Thus, all flies were in the same genetic background before experiments began. All stocks and experimental flies were maintained in plastic vials or bottles on sugar-yeast-molasses medium with *ad libitum* live yeast granules at 25°C on a 12∶12 hr light dark cycle (except where specified). We used a standard density method to rear flies. First instar larvae were picked from petri dishes containing an agar-grape-juice laying medium and placed in batches of 150 into plastic bottles containing 50 mL of food.

### Reproductive Success of SAA and Control Males and Females

We measured male mating success by introducing a single virgin wild-type female (N = 28) into a vial containing a virgin control male and a virgin SAA male of matched age. Experiments were conducted in light or in dark under red-light (*D. melanogaster* cannot see red light). We recorded which male mated first. To assay the post-copulatory competitive ability of SAA and control males, we conducted tests of sperm defense (P_1_, the paternity share of the first male to mate with a female) and sperm offense (P_2_, the paternity share of the second male to mate with a female). The competitor males and the females were homozygous for the *sparkling^poliert^* (*spa^pol^*) mutation [37]. *spa^pol^* homozygotes posses a distinct eye phenotype which allows for easy visual determination of paternity. All flies were 3–5 days post-eclosion at the time of first mating. To assay P_1_, single virgin *spa^pol^* females were first mated to either a SAA or control male, and then mated to a single *spa^pol^* male 24 hours after this initial mating. Females were then allowed to oviposit individually in vials for 24 hours. Offspring from these vials were assayed for paternity (SAA, N vials = 23; control, N vials = 27). The P_2_ assay was identical except that the matings were reversed: the first mating was conducted with *spa^pol^* males, and the second mating with either a SAA or control male (SAA, N = 21; control, N = 16). To measure offspring production of females we placed 5 3-day old virgin SAA, heterozygote or control females in vials with 5 virgin SAA or control males of the same age (i.e., 6 cross combinations). Flies were transferred to fresh vials every 2 or 3 days until day 10 when they were separated into pairs of 1 male and 1 female and transferred to fresh vials for 24 hrs. Eggs oviposited over the 24 hrs were counted. 14 days later the eclosed offspring were counted and scored for eye colour.

### Experimental Evolution Populations

Flies for the 1^st^ generation P1–P8 populations were virgins generated from crosses between heterozygote females and SAA and control males. P1–P4 initially contained 9 SAA and 81 control males, and 100 control females (i.e., 3% SAA bearing X-chromosomes, 97% control X-chromosomes). Initial numbers of SAA and control males, and SAA, heterozygote and control females were, respectively, P5) 44, 56, 4, 42, 54 (i.e., 31% SAA X-chromosomes); P6) 65, 35, 12, 56, 31 (i.e., 48% SAA); P7) 81, 19, 29, 57, 14 (i.e., 65% SAA); P8) 94, 6, 64, 33, 2 (i.e., 85% SAA). These proportions were calculated based on selection at Hardy-Weinberg equilibrium using rudimentary fitness estimates (calculated when P5–P8 were set up) for each genotype (1 for SAA and 0.55 for control males, 0.388 for SAA females, 0.9 for heterozygote females, and 1 for control females).

Adult flies were placed in a 4.5 L plastic cage containing a food bottle, which was replaced every 2 or 3 days. After 8 days eggs were collected for propagation of the subsequent generation. 13 days later (i.e., typically 2–3 days after the majority of flies had eclosed, allowing ample time for development), offspring were counted and eye colour recorded to determine genotypes. The proportions of genotypes were calculated and the next generation of 100 males and 100 females was established for each population based on these proportions, rounded to the nearest integer. During photophase we made a total of 62 spot-check mating observations on P1–P4 – over generations 1, 3–7, 9, 11, 12 and 15 – to estimate the relative mating success of SAA and control males in the population cage environment.

### Mathematical Modeling

We modeled the spread and maintenance of the SAA using a standard population genetic approach. We consider a population of SAA and control genotypes. At each generation the number of matings between males and females of each genotype combination was calculated based on the frequency of each male and female genotype in the population and the empirically-derived advantage for the SAA allele in males. This SAA male advantage was calculated by taking the mean mating success of males during light phases in the experimental environment (Figure 2b), and adjusting it for the hours of light in the light-cycle (e.g. 12∶12) and the proportion of matings expected to occur in light *vs* dark (0.402∶0.598, light∶dark, calculated from references [33], [34]). The frequencies of each male and female genotype for the following generation were then calculated based on the mean number of surviving offspring of each genotype produced by each type of mating (i.e., male-female genotype combination) observed in our experiments (Table S1). We set the initial genotype frequencies at generation 1 to be the initial frequencies used in the experiment and determined the equilibrium SAA frequency after 1000 generations.

To generate confidence intervals around the predicted equilibria, we introduced the random selection of 300 offspring genotypes from all those generated to make up the next generation. This step mirrors the experimental procedure, in which 300 larvae were taken each generation from all those available. The total number of offspring generated (from which 300 were selected) varied with each generation and with the parameter values used, and was typically 2500–5400. Each run of this simulation model generated new frequencies of the SAA at each generation. We performed 100 runs of the model with each set of parameter values and then calculated at each generation the mean, standard deviation, and 95% confidence interval for SAA frequency.

### Statistical Analysis

Data were analysed using R and JMP v9. SAA male mating advantage was calculated using chi square tests on the total number of observed SAA-male and control-male mating opportunities taken as a proportion of the total number of potential mating opportunities (i.e., a product of the frequency of SAA in each generation and the total number of mating observations each generation). P1 and P2 data for the sperm competitive ability assays could not be satisfactorily normalized and so were analyzed using Wilcoxon signed ranks tests. Analyses using parametric methods (i.e., t-tests on data that was Box-Cox transformed) produced qualitatively similar (i.e., non-significant) results. Female fitness costs of bearing the SAA were analyzed using a generalized linear model (GLM) with Poisson error distribution on the total number of offspring resulting from each of the six combinations of parental crosses. Father (2 level factor), mother (3 level factor) and their interaction were specified as fixed effects. SAA frequency data in P1–P8 and in the light/dark lines were analyzed with generalized linear mixed-effects (GLMM) models. To account for replicate lines and for repeated measures across generations, line within generation was specified as a random effect in all GLMM models. Generation and, where appropriate, ln generation, light manipulation or initial SAA frequency were specified as fixed effects. To analyze the change in SAA frequency in P1–4 in more detail we conducted a segmented regression. We partitioned the data based on the observation that the change in SAA frequency appeared to follows 3 distinct phases of increase, decrease, and plateau. Thus, we tested for changes in SAA frequency between generations 1–6, 6–10, and 10–16.

## Supporting Information

Figure S1 Proportion of offspring sired by SAA and control males following post-copulatory competition (a) Paternity share of the first male to mate with a female (b) Paternity share of the second male to mate with a female.
(TIF)

Table S1 Number of offspring of each genotype produced when a single female (SAA, heterozygous or control), mated to either control or SAA males, was allowed to lay eggs over a 24 hr period.
(DOCX)

Table S2 Results from a generalized linear model with Poisson error distribution of the number of offspring produced when a single female (SAA, heterozygous or control), mated to either control or SAA males, was allowed to lay eggs over a 24 hr period.
(DOCX)
