# Supplementary figures and images for: Experimental Evolution of a Novel Sexually Antagonistic Allele
Source: PLoS Genet. 2012 Aug 30;8(8):e1002917. doi: 10.1371/journal.pgen.1002917 (PMC3431318; doi:10.1371/journal.pgen.1002917)

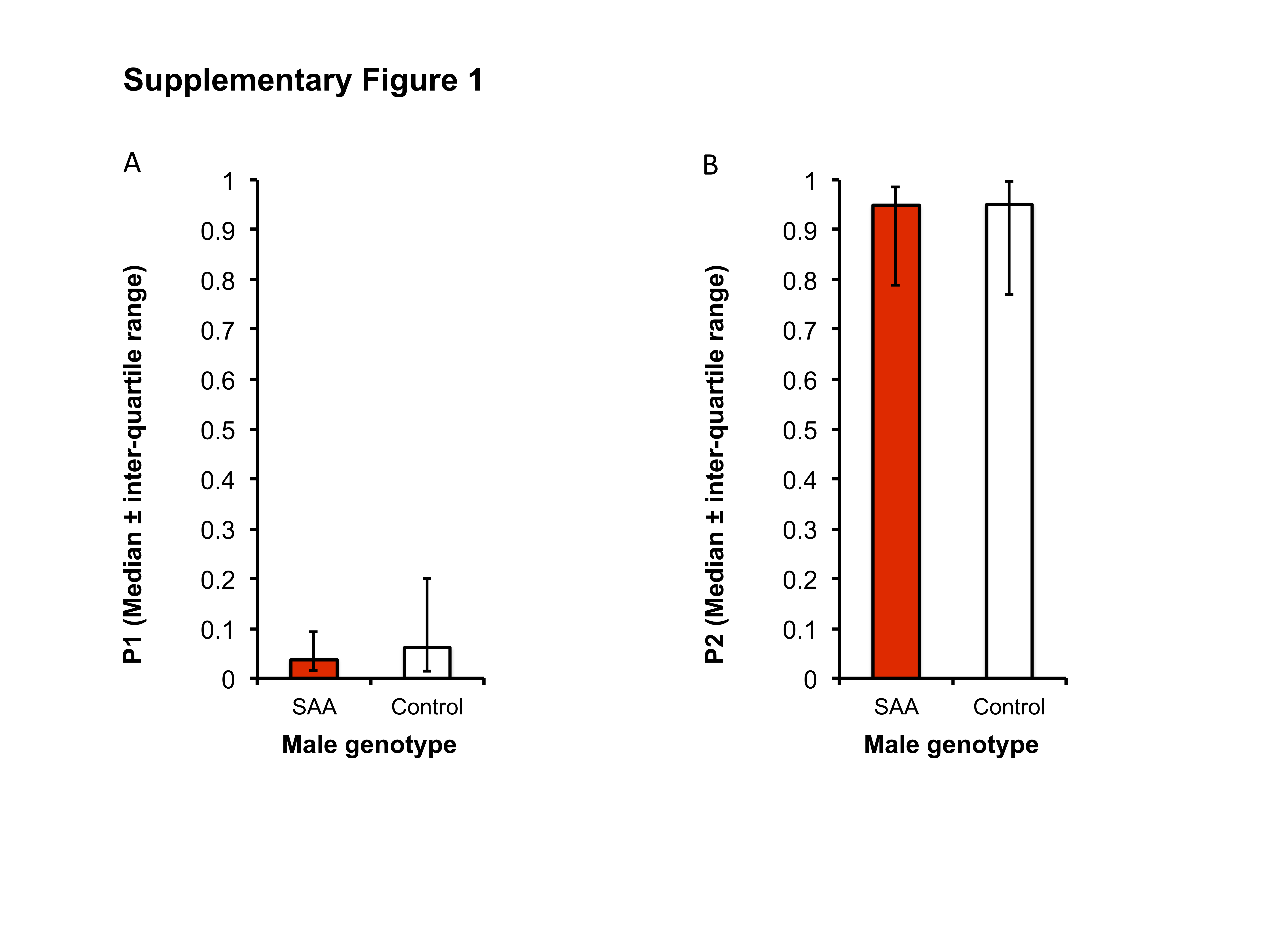

Supplement: Figure S1 — Proportion of offspring sired by SAA and control males following post-copulatory competition (a) Paternity share of the first male to mate with a female (b) Paternity share of the second male to mate with a female. (TIF) [file pgen.1002917.s001.tif]
