# Supplementary material for: Experimental Evolution of a Novel Sexually Antagonistic Allele
Source: PLoS Genet. 2012 Aug 30;8(8):e1002917. doi: 10.1371/journal.pgen.1002917 (PMC3431318; doi:10.1371/journal.pgen.1002917)
Supplement: Table S1 — Number of offspring of each genotype produced when a single female (SAA, heterozygous or control), mated to either control or SAA males, was allowed to lay eggs over a 24 hr period. (DOCX) [file pgen.1002917.s002.docx]

**Table S1**

| Male parent | Female parent | Offspring sex | Offspring genotype | Mean # offspring | S.E. mean | Mean total offspring | S.E. total offspring |
| --- | --- | --- | --- | --- | --- | --- | --- |
| Control | Control | Female | Control | 28.2 | 1.9 | 54.0 | 3.1 |
| Control | Control | Male | Control | 25.9 | 1.6 |  |  |
| Control | Het | Female | Control | 12.9 | 0.7 | 49.1 | 2.0 |
| Control | Het | Female | Het | 12.7 | 0.7 |  |  |
| Control | Het | Male | Control | 12.8 | 0.8 |  |  |
| Control | Het | Male | SAA | 10.8 | 0.6 |  |  |
| Control | SAA | Female | Het | 11.2 | 2.4 | 20.2 | 4.2 |
| Control | SAA | Male | SAA | 8.9 | 1.8 |  |  |
| SAA | Control | Female | Het | 20.6 | 2.7 | 40.7 | 5.3 |
| SAA | Control | Male | Control | 20.2 | 2.8 |  |  |
| SAA | Het | Female | Het | 12.1 | 1.2 | 41.9 | 3.8 |
| SAA | Het | Female | SAA | 10.1 | 1.0 |  |  |
| SAA | Het | Male | Control | 10.6 | 1.1 |  |  |
| SAA | Het | Male | SAA | 9.0 | 0.9 |  |  |
| SAA | SAA | Female | SAA | 13.8 | 1.5 | 25.3 | 2.4 |
| SAA | SAA | Male | SAA | 11.5 | 1.0 |  |  |
