# Supplementary material for: Experimental Evolution of a Novel Sexually Antagonistic Allele
Source: PLoS Genet. 2012 Aug 30;8(8):e1002917. doi: 10.1371/journal.pgen.1002917 (PMC3431318; doi:10.1371/journal.pgen.1002917)
Supplement: Table S2 — Results from a generalized linear model with Poisson error distribution of the number of offspring produced when a single female (SAA, heterozygous or control), mated to either control or SAA males, was allowed to lay eggs over a 24 hr period. (DOCX) [file pgen.1002917.s003.docx]

**Table S2**

| **Fixed Terms** | **Parameter Estimate** | **SE** | **DF** | **F** | **P** | |
| --- | --- | --- | --- | --- | --- | --- |
| Male SAA | 3.56 | 0.04 | 1, 168 | 0.69 | 0.4063 | |
| Control | 3.64 | 0.04 |  |  |  | |
| Female SAA | 3.13 ^a^ | 0.07 | 2, 168 | 55.4 | **<0.0001** | |
| Het | 3.82 ^b^ | 0.06 |  |  |  | |
| Control | 3.85 ^b^ | 0.06 |  |  |  | |
| Male*Female SAA*SAA | 3.74 | 0.07 | 2, 168 | 5.27 | 0.0717 | |
| SAA*Het | 3.55 | 0.06 |  |  |  | |
| SAA*Control | 3.49 | 0.06 |  |  |  | |
| Control*SAA | 3.44 | 0.07 |  |  |  | |
| Control*Het | 3.64 | 0.06 |  |  |  | |
| Control*Control | 3.70 | 0.06 |  |  |  | |
| Note: GLM fitted with a Poisson error distribution and correction for overdispersion. Significant values are shown in bold. ^a,b^ denotes significant LS Means differences between levels. | | | | | |  |
